# Danish Medical Institutions

**Published:** 1904-01-09

**Authors:** 


### DANISH MEDICAL INSTITUTIONS.

(*Concluded from page* 138.)

### III.—A CRIPPLE'S SCHOOL AND MEDICAL MACHINERY.

I had long heard of the school for cripples carried on in Copenhagen by Fröken Thora Fiedler, and was very glad to be at last able to visit it. I was warned that there was no one in the establishment who could speak English, so took with me an English-speaking Danish friend. As a matter of fact, the surgeon who has control of the hospital from which the school arose spoke admirable English and knew all the orthopædic hospitals in London. I was permitted to be present during the hour when he saw patients, and though I could not understand what he said to them, I could admire the deft way in which he handled each case, and the perfect confidence which all his patients, even nervous little children, seemed to have in him. He even offered to let me be present when he performed the operation for reducing a congenital dislocation of the hip-joint, made famous by Dr. Lorenz, but a glance at the face of my Danish friend, who had no taste for surgery, compelled me to decline.

So we proceeded to the school. School is perhaps too limited a name for the institution, for many of the pupils who learn some kind of work within its walls remain there to practise the trade they have acquired. And as it was holiday time and the children were not in residence, it was the older inmates whom I saw at work. I speak, therefore, rather of results than of methods. The school—which is an old mansion house, presenting a bare front to the street, but within built round the four sides of a sunny courtyard—contains workshops where cripple girls are taught to sew and to weave, and boys and men to make shoes, do carpentering, and even elaborate furniture-making and wood-carving. Much of the furniture in the rooms occupied by the matron and her assistants has been made by the inmates, and it is both substantial and elegant. Where the pupils are afflicted in the lower limbs only, the task of teaching them is comparatively simple. But a very large proportion of those whom I saw had lost either the right or the left arm. One girl, whose left arm had been amputated a little below the elbow, put a fine needle in the crook of the elbow and holding it thus threaded it deftly, and went on with her sewing, holding the seam over the stump of the arm where an ordinary person would hold it between thumb and forefinger. Another sewed with the left hand, the right having been amputated. A clamp, which fixed the garment to be sewed to the table was the only special assistance which these girls had in their work; for the rest they had simply been carefully trained to use what limbs they had left. In the weaving-room the majority of the workers were one-handed, but this seemed to cause them inconvenience only when it was necessary to join on a new thread. At other times they worked as fast, or nearly so, as an ordinary hand-loom weaver. One girl who had only her left hand to work with—the right hung paralysed before her—was engaged on an elaborate piece of work which practically combined embroidery with weaving, as needlefuls of different coloured wools were placed at intervals along the web, and the weaver worked them in between the strokes of the shuttle. The work which all these weavers were doing was far more difficult, and made greater demands on eye, hand, and brain, than the ordinary work of a fully-equipped weaver in a factory.

The same may be said of the work on the men's side. One-armed men and boys, if they had but a stump or a hook wherewith to hold their work, were planing and sawing, and a very beautiful panel in carved wood, showing delicate work in high relief, with much undercutting, was the work of a man with one hand. Another showed with great pride an elaborate jointed wooden hand, the making of which he had just completed. The shoemaker's shop really includes a very considerable surgical instrument factory. Here are made the high boots, the firm corsets, and the trusses required by the patients of the hospital, and the doctor who prescribes them has thus a better assurance that his measurements will be accurately carried out. The philanthropic and economic value of this school for cripples is undeniable, and there is much room for work of this sort in England. The institution has now 200 pupils. If Copenhagen, with less than a quarter of a million inhabitants, can send so many there, what number must there be in London who are as yet untrained to make use of such limited powers as they have!

Another institution which interested me greatly was the "Medico-Mechanic Institute." I had been told it was a place where massage was given by machinery, but the doctor in charge smiled at this description. Massage is an art, he said, and cannot be so applied. One or two of the instruments in the institute—which looks rather like a mediæval torture-chamber, though it is a home of healing—do, however, give some massage movements. The majority are meant to relieve rheumatic stiffness of the joints, which is very common in the damp climate of Denmark. In some the machine does all the work, in others the patient assists. There are also several kinds of vibration machines, and some for expanding the lungs and compelling diaphragmatic breathing. In all the resistance is regulated by means of weights, and the whole treatment is under constant medical supervision. No one movement is continued throughout a whole séance, and the doctor's prescription, which the attendants must see carried out, commands that Christian Jorgensen, for example, shall exercise for ten minutes on machine No. 6, then make twenty movements on No. 10, go on for five minutes to No. 13, and after five minutes' rest return to No. 6 again. The amount of force to be applied or resistance to be overcome is also carefully stated. The value of the method dwells largely in this continuous medical supervision and regulation. In unskilled hands the treatment might be useless and even dangerous; with proper control it is healthful and, as I can testify, exhilarating. The charge made for an hour's treatment daily is 25 kroner a month.

